# The creation of a confidence scale: the confidence in managing challenging situations scale

**DOI:** 10.1177/1744987120979272

**Published:** 2021-02-18

**Authors:** Pauline Walsh, Patricia Owen, Nageen Mustafa

**Affiliations:** Pro Vice Chancellor and Executive Dean, Faculty of Medicine and Health Sciences, 4212 Keele University, UK; Head of School of Nursing and Midwifery, 4212 Keele University, UK; Research Associate, School of Nursing and Midwifery, 4212 Keele University, UK

**Keywords:** challenging situations, confidence scale, nursing, resilience, self-confidence, self-efficacy

## Abstract

**Background:**

Self-confidence and self-efficacy are vital psychological constructs that can affect a student’s performance.

**Aims:**

To measure the level of confidence in nursing students in managing challenging situations in clinical practice settings.

**Methods:**

In order to develop the scale three focus groups were conducted: with registered nurses, third year student nurses and service users. Focus group frameworks included: challenging behaviours, managing challenging situations and preparing students to manage challenging situations. Themes in relation to challenging situations that emerged from the focus groups, in conjunction with Nursing and Midwifery Council standards and expert discussions were used to create the confidence scale. The Confidence in Managing Challenging Situations Scale consists of two parts with 21 items in total. Both parts were measured by way of a five-point Likert scale. The scale was utilised to determine the level of confidence of students both pre and post a teaching intervention.

**Results:**

The confidence in managing challenging situations scale had good internal consistency, with a Cronbach’s alpha coefficient reported of 0.86. Exploratory factor analysis was used to support the scale validation process.

**Conclusions:**

The Confidence in Managing Challenging Situations Scale is a successful measure of confidence for nursing students in healthcare settings. It can be applied in alternative healthcare settings for the identification of confidence levels in those student nurses learning in care settings.

## Introduction

### Background

Self-confidence and self-efficacy have been reported as vital psychological constructs that can affect a student’s performance (Craven et al., 1991; Hay et al., 1997; Kukulu et al., 2013).

The self-efficacy theory of Bandura (1986) suggests that confidence is enhanced by four main factors: successful performances (competence), vicarious experience, verbal persuasion (including praise and encouragement), and physiological feedback. Furthermore, Bandura and Locke (2003) have found that one’s own belief in self-efficacy can significantly impact on confidence in motivation and performance. In terms of developing confidence, Bandura’s theory proposes that ‘mastery experiences’ are crucial for the development of self-efficacy (Bandura, 1977). Perry (2011) states that self-confidence is ‘a self-perceived measure of one's belief in one's own abilities, dependent upon contextual background and setting’ (Perry, 2011: 219).

Bandura found that there is no ‘one measure fits all’ (Bandura, 2005: 307) in terms of measuring self-efficacy, but that it must be linked to the area of functioning. It is believed that when trying to apply the same measure to different situations, items in the measure are cast in general terms and so may be dissociated with the actual state being measured (Bandura, 2005). To avoid any ambiguity about what is actually being measured and the situational demands of a task, Bandura (2005) states that self-efficacy scales should be tailored to the ‘object of interest’ and modified according to the context within which it is being used. It is important then to identify the object of interest when developing self-efficacy scales. In the case of nursing it has been widely reported that nurses have to contend with challenging situations (Huston, 2013; Wangensteen et al., 2008), and that student nurses in particular face a range of particular circumstances that they may find challenging (Cooke, 1996).

This paper sets out to outline the development of a confidence scale that measures the level of confidence in nursing students. However, it is suggested that the tool that has been created could be modified and applied to a range of disciplines and users accordingly. The overarching aim of this tool is to measure the level of confidence in nursing students in managing challenging situations in clinical practice settings. The level of confidence was measured both pre and post a teaching intervention that was developed as part of an undergraduate university nursing programme.

Evidence-based searches using PsychINFO, EBSCO and Web of Science were used to identify research surrounding confidence in student nurses. Key words included: confidence, self-efficacy, nursing, student learning and teaching. Due to the limited number of papers that have been published about confidence in nursing students, a date range was not selected. Papers were then individually critiqued for rigor and relevance using a three-stage approach of appraisal of title, appraisal of abstract and then appraisal of the whole paper. In total, 275 resources were identified as being relevant to the topic area. Through appraisals this was then reduced to 106 then 44.

### Confidence in nursing

Confidence in nursing is not a new phenomenon (Farrand et al., 2006). It has often been acknowledged as a crucial component of effective nursing practice. This suggests that developing confidence is vital in the education and training of nursing students to prepare them better for the clinical setting in the ‘real world’.

Within the United Kingdom, all approved education institutions and their clinical placement provider partners must adhere to the requirements set out by the Nursing and Midwifery Council (2018) and implement them within pre-registration nursing education. Standards for pre-registration nurse education include proficiencies that are delivered through NMC-approved 3-year undergraduate degree programmes which include equal amounts of learning taking place within the university and practice placement settings.

Part of the skill set that is thought to be required by nursing students is having the confidence to transition learning from theoretical teachings to clinical practice in the ‘real world.’ Lundberg (2008) reported that student nurses who lack confidence are less able to deal with situations that they find difficult in a clinical setting. The author further asserted that it is crucial that nurse educators work to instil confidence in nursing students through a ‘confidence rich learning environment’ to help better prepare them for work in a clinical setting (Lundberg, 2008). Teaching strategies have been found to help shape the development of student confidence in healthcare professions (Blum and Gordon, 2009; Jordan and Farley, 2008). Stump and colleagues (2012) state that self-efficacy has a great impact on a nurse’s performance and, consequently, the wellbeing of the patient. In a study which investigated transition of newly qualified nurses in a UK hospital, confidence and resilience were identified by participants as being major factors in the preparation of newly qualified nurses as they joined the workforce (Whitehead et al., 2016). Therefore, it is important for nurse educators to assess students’ ability in terms of confidence and self-efficacy.

### Confidence/self-efficacy scales

In order to measure levels of confidence and self-efficacy in student populations, scholars have devised or used existing scales. For instance, Van Horn and Christman (2017) utilised the Clinical Skills Self-Efficacy Scale (CSES) to evaluate the confidence levels of junior and senior baccalaureate nursing students in performing selected skills after they completed their skills laboratory courses. The CSES was developed to explore the relationship between self-efficacy and clinical skills acquisition. Their results showed that junior students scored significantly lower in terms of self-efficacy than the average score for seniors (Van Horn and Christman, 2017). Van Horn and Christman (2017) assert that the reason juniors scored lower than seniors was due to fewer opportunities being available to perform clinical skills. Zimmerman and Kitsantas (2005) developed the Self-Efficacy for Learning Form (SELF) to assess high school students’ feelings towards coping with challenging academic problems or contexts. The scores on the scale were found to have a high level of internally consistent reliability (Cronbach’s alpha 0.96). This scale was then modified by Zimmerman and Kitsantas (2007) and applied to university students. They developed an abridged form of the scale entitled SELF-A. Statistical analysis showed that scores on the SELF-A were high in their reliability (Cronbach’s alpha 0.98). This indicates that scales may be modified and applied to other populations.

Stump and colleagues (2012) have reported that commonly used measures of self-efficacy that are based on the classic true score theory as the theoretical basis (e.g. Arnold et al., 2009; Brown and Chronister, 2009; Cheraghi et at., 2009; Clark et al., 2004) have been found to have limitations. For example, limitations are identified ‘when test or survey items are analysed and data are interpreted, such as generation of a single reliability estimate for an entire scale and sample dependent score interpretation’ (Stump et al., 2012: 150).

### Scale development

Overall, a range of scales has been developed to measure levels of confidence among different populations (Boateng et al., 2018). The literature review failed to identify a confidence scale that specifically focused on nursing students meeting challenging situations in practice. Therefore, for the purpose of the present study, a confidence scale was designed to capture the confidence levels of nursing students when dealing with challenging situations on an undergraduate nursing course pre and post a teaching intervention.

The ‘three phases and nine steps of scale development and validation’ proposed by Boeteng and colleagues (2018) were partially utilised to inform the creation of the present confidence scale. Boeteng et al. (2018) proposed three phases to creating a rigorous scale, including: item development, scale development and scale evaluation. Within these phases they describe steps that need to be taken, these include: identification of the domain and item generation, consideration of content validity, turning individual items into a measuring construct, pre-testing questions, sampling and survey administration, item reduction, extraction of latent factors, tests of dimensionality, tests of reliability and tests of validity. Reliability may be described as the degree of consistency exhibited when a measurement is repeated under identical conditions (Porta, 2008). In order to assess the reliability of a scale Cronbach’s alpha may be used (Cronbach, 1951). Other statistics used to assess the reliability of scales include: ordinal alpha (Gadermann et al., 2012; Zumbo et al., 2007), test–retest reliability (Raykov and Marcoulides, 2011), McDonald's omega (McDonald, 1999), Raykov's rho (Raykov and Marcoulides, 2011) or Revelle's beta (Revelle, 1979; Revelle and Zinbarg, 2009), split-half estimates, Spearman–Brown formula, alternate form method (coefficient of equivalence) and interobserver reliability (Raykov and Marcoulides, 2011). Boeteng and colleagues (2018) reported that Cronbach's alpha is predominantly used to assess the reliability of scales (Cronbach, 1951; Raykov and Marcoulides, 2011) and that it has received wide-ranging support from scholars.

In addition, exploratory factor analysis (EFA) has been used to determine the construct validity of an instrument during the initial development stages. In particular, EFA may be used to test the psychometric properties and validity of scales when following general guidelines of scale development (Hinkin, 1998; Korlen et al., 2018).

For example, when a set of items designed to measure a construct has been created, EFA can be utilised to examine the underlying dimensionality of the item set (Worthington and Whittaker, 2006). It has been asserted that by using EFA it allows items to be related to any of the factors underlying responses, so that items that do not measure an intended factor or those that simultaneously measure multiple factors can be identified. These factors may not be accurately measuring the construct they are intended to, and so can be eliminated from the scale (Worthington and Whittaker, 2006).

Taherdoost and colleagues(2014) affirm that the main objectives of EFA are:

- reduction of the number of factors (variables);
- assessment of multicollinearity among factors which are correlated;
- unidimensionality of constructs evaluation and detection;
- evaluation of construct validity in a survey;
- examination of factors (variables) relationship or structure;
- development of theoretical constructs;
- prove proposed theories.

Although there is some debate over the sample size needed for factor analysis (Hogarty, et al., 2005; Tabachnick and Fidell, 2007) it has been suggested that data itself can determine the adequacy of sample size. For instance, high communalities without cross-loadings, plus several variables loading strongly on each factor suggest that EFA can be used as an accurate measure for validity despite a small (under 100) sample size (Costello and Osborne, 2005).

### Likert scale

There are a number of rating scales that have been developed to measure an individual’s response based on their attitude. The most widely used psychometric tool in educational and social research is the Likert scale (Joshi et al., 2015; Likert, 1932). This type of measure was chosen to analyse levels of confidence in the scale as it allows the respondent to give degrees of opinions or no opinion rather than simply answering ‘yes’ or ‘no’. By utilising Likert scales all participants are able to remain anonymous, as little or no identifiable information is given due to the set options provided by the scale. By offering anonymity social desirability bias is reduced (Joinson, 1999). The choice to use the Likert scale was in part related to the subject matter being investigated and the way in which the data would be evaluated. This is partially supported by Joshi and colleagues (2015) who found that the ‘construct of research instrument can be derived from objectives of study and objectives are the operational form of theoretical construct of phenomenon under inquiry’.

The creators of the scale reviewed several papers that discussed the properties of Likert scales (see Hartley and Betts, 2010; Krosnick and Fabrigar, 1997 and Oppenheim, 2000).

The limitations of the scale were also identified and acknowledged. These included: asking about more than one thing in one question (causing difficulties for the respondents; Hartley, 2014), using several different scales in one questionnaire and when the layouts of these scales vary (Betts and Hartley, 2012), people may avoid choosing the “extreme” options on the scale and want to be more neutral even though the extreme would be the most accurate (Albaum, 1997).

## Methods

Ethical approval was given by the university for the project to take place and informed consent was obtained from all participants. In developing the scale of confidence, three focus groups were conducted. The purpose of these focus groups was twofold. The first was to help inform the intervention utilised within this study. The second was to facilitate the creation and development of a confidence scale using expert opinion (QSR software NVivo 10 was used to conduct a thematic analysis). Three participant groups of: registered nurses, third year student nurses and service users were identified as pertinent to the area being explored and therefore a focus group for each participant group was set up (see Table 1).

**Table 1.** Focus groups.

Focus group	Participants	No. of participants
1	Nurse practitioners	3
2	Service users/carers	14
3	Third year nursing students	5

The focus-group framework for discussion centred on the three key areas of definitions of: challenging behaviours, managing challenging situations and preparing students to manage challenging situations. All focus groups asked participants to consider their own experiences of either working in a hospital environment or using the services provided by hospitals. The focus groups identified a range of issues that students may face with the top three from each outlined below. One of the issues that emerged from all groups, although described in differing ways, was the extent of the student’s ability to challenge practice and how communication took place between people in each group. For example, one student stated: ‘she was really patronising and I felt there’s ways and means of talking to people and it was the fact she did it in front of patients that really got me’ (SN1). Another student said:

she was very – when she gave feedback it wasn’t constructive; it was just negative. There was nothing constructive about it. ‘Well, you didn’t do that very well. You didn’t do that’, and that started to chip away at my confidence as well. (SN 2)

Both registered practitioners and service users or carers commented on the environment in which students provide care as being one that is challenging; for example, a service user commented that ‘the nursing staff are just too busy’ (SU 1). While a registered nurse indicated that ‘environment that you’re working in. If you are working in an emergency situation or if you are in – that is quite challenging’ (RN 1).

Overall, a number of key challenging situations emerged from the focus groups, the literature and expert discussions resulting in 12 being included within the confidence scale.

### Scale creation

Following design, measurement and instrument development the confidence scale was created by Walsh and Owen (2018). This was named the Confidence in Managing Challenging Situations (CMCS) Scale. This scale was based both on themes that had arisen from three focus groups and the NMC standards that students have to demonstrate competence in, in order to achieve registration as a nurse (Nursing and Midwifery Council, 2010).

The scale was developed to determine the level of confidence of students both pre and post receiving the teaching intervention. Teaching and training, in addition to the existing curricula surrounding resilience in nursing, was implemented into year 2 of the nursing and midwifery course at a UK University.

In particular, the teaching intervention consisted of a combination of practice-based learning and university-based learning with two workshop days. The first day took place at the beginning of the course and was followed by a period of clinical placement. The second day occurred after the first placement had concluded and was followed by a second period of clinical placement.

The existing curriculum already included two sessions on resilience and building resilience in practice, which were undertaken as normal. The workshops were planned to ensure that students were active in their learning, and the content addressed those areas students might find challenging as evidenced from the literature and the focus-group data. Teaching was classroom based with the exception of simulation sequences that were utilised to replicate real-life scenarios using professional actors in a lab-based setting.

Day 1 of the intervention consisted of teaching on the following subjects: building resilience into practice, advocacy, empowerment, examining challenging situations and managing conflict. Study objectives were split into three areas. The first was to identify core aspects of resilience. The second was to examine and define the concept of challenging situations. The third was to develop an understanding of mindfulness, assertive behaviour, advocacy and managing conflict.

The study objectives of day 2 included: identifying key elements necessary when advocating for a patient and discussing the principles involved in managing learning in potentially challenging environments. Throughout this students were required to discuss individual professional development reflections and examine strategies for managing an unexpected situation with their teaching groups that they had been assigned to. All discussions were facilitated by a teacher.

**Figure 1.**
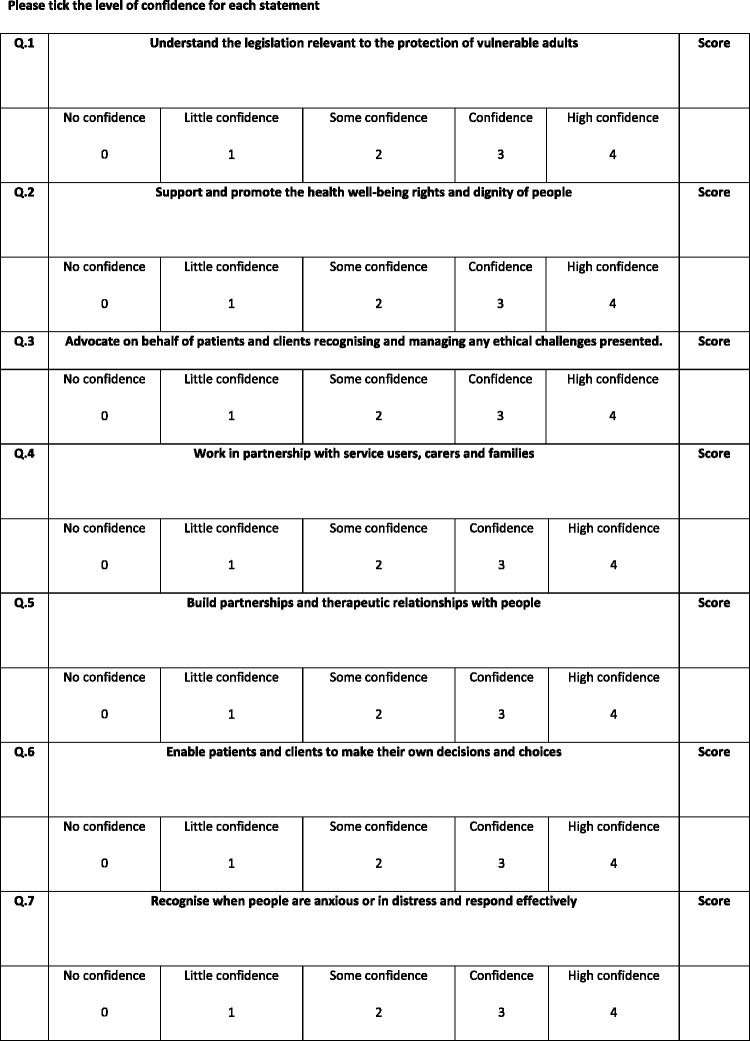

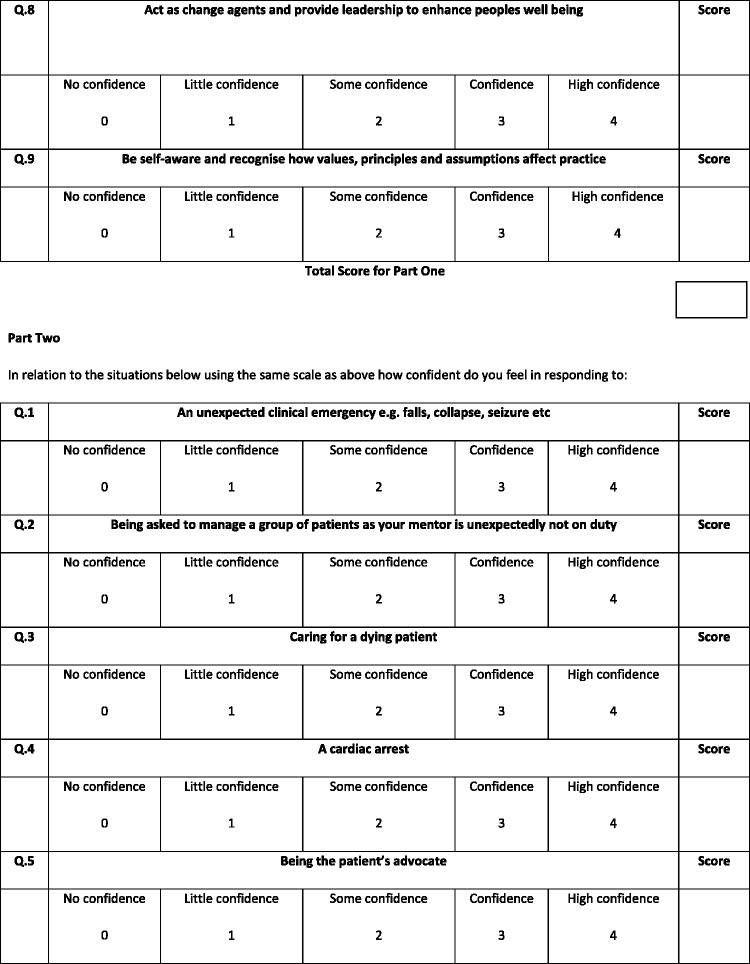

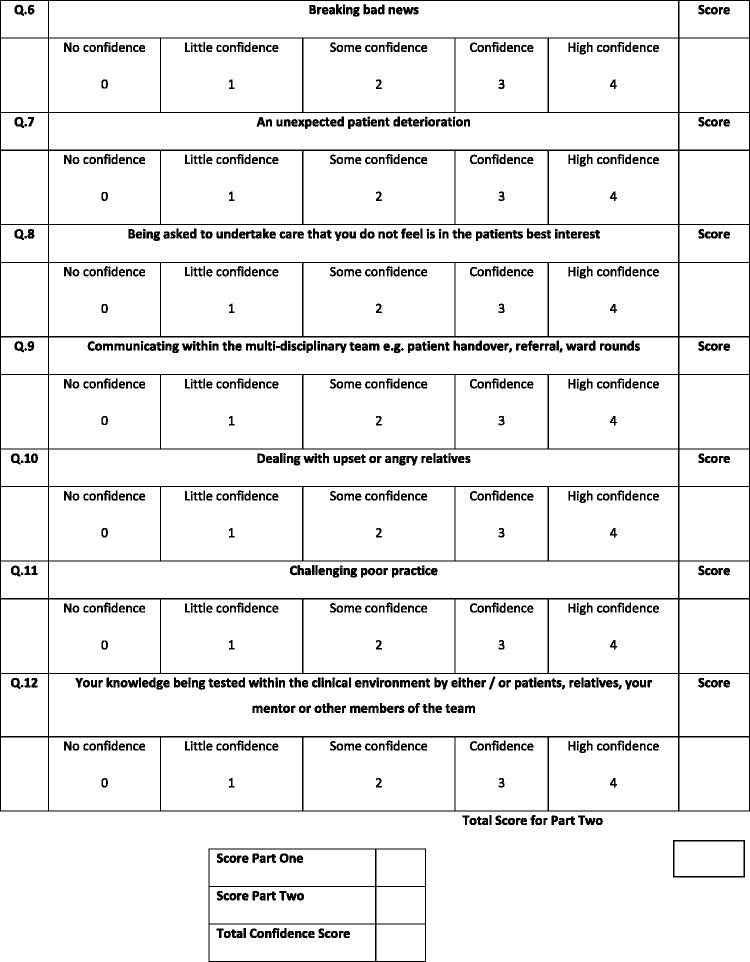
The Confidence in Managing Challenging Situations Scale.

This scale consisted of two parts. The first contained nine items which were related to the NMC standards of competency at the time (NMC, 2010) and the second contained 12 items. Both parts were measured by way of a five-point Likert scale from ‘no confidence’ to ‘high confidence’ (see Figure 1).

Interpretations of the scores were given to participants on completion of the scale on both days. There were three ranges of scores indicating either low confidence (0–29), confident (29–60) or high confidence (61–84).

## Results

In total, 70 students completed the CMCS scale pre and post the teaching intervention.

In order to measure the internal consistency of the CMCS scale a Cronbach's alpha was carried out. Cronbach's alpha assesses the internal consistency of the scale items; that is, the degree to which the set of items in the scale co-vary, relative to their sum score. An alpha coefficient of 0.70 has often been regarded as an acceptable threshold for reliability; however, 0.80 and 0.95 is preferred for the psychometric quality of scales (Cronbach, 1951).

Findings showed that the CMCS scale had good internal consistency, with a Cronbach’s alpha coefficient reported of 0.86 (see Table 2).

**Table 2.** Reliability statistics.

Cronbach's alpha	Cronbach's alpha based on standardised items	No. of Items
0.863	0.870	21

Exploratory factor analysis was also conducted to establish the statistical validity of the scale. The 21 items of the CMCS scale (Walsh and Owen, 2018) were subjected to principal components analysis using SPSS version 24. Prior to performing principal components analysis, the suitability of data for factor analysis was assessed. Inspection of the correlation matrix revealed the presence of many coefficients of 0.3 and above. The Kaiser–Meyer–Olkin value was 0.79, exceeding the recommended value of 0.6 (Kaiser, 1970, 1974) and Bartlett’s test of sphericity (Bartlett, 1954) reached statistical significance, supporting the factorability of the correlation matrix. Principal components analysis revealed the presence of six components with eigenvalues exceeding 1, explaining 28.7%, 8.4%, 8.2%, 6.2%, 5.8% and 5.0% of the variance, respectively. An inspection of the scree plot revealed a clear break after the first component and so justified the factor structure. Using the scree test of Catell (1966), it was decided that no further factor analysis was necessary (a sharp flattening of the eigenvalues at the second component suggests just 1 factor).

## Discussion

This paper describes the development, validation and implementation of a confidence scale entitled the CMCS scale (Walsh and Owen, 2018). The aim of the creators was to facilitate the development of a new, valid and reliable scale to be used in the nursing sector; in particular, to measure the level of confidence in nursing students in managing challenging situations in clinical practice settings.

In the first instance the scale has been used to measure the confidence levels of healthcare students before and after a teaching intervention (in press). The scale has been based on research obtained through focus groups including: health professionals, students and service users. Previous confidence scales designed to measure the confidence of health professionals in challenging situations are scarce. However, the scales that have been devised to measure self-efficacy in student populations have been validated using Cronbach's alpha (Zimmerman and Kitsantas, 2005, 2007). This method was used to validate the CMCS scale and the findings showed good internal consistency. Exploratory factor analysis showed support for the use of the question items to measure confidence in challenging situations, as suggested by the scale authors (Walsh and Owen, 2018). Therefore, we can conclude that the CMCS scale is a reliable and valid tool that may be used to collect information on students’ confidence. Initially, the tool has been utilised to obtain data from students in the field of healthcare. However, this tool could be used with other healthcare professionals following training interventions or could be modified to be used with anyone who works in a healthcare setting, or those whose employment involves any form of patient contact. In addition, the CMCS scale could be used as a self-assessment tool and part of an educational activity across the health education sector. By using this tool educators can help to ensure that students are developing in areas that may prepare them for clinical practice.

Overall, the CMCS scale is straightforward to use, is based on real-world concepts, is measured using a Likert scale, has good internal consistency, reliability and validity, while each item is meaningful independently. The scale provides a summary of the implications based on the score received giving an immediate indication of an individual’s level of confidence. This can then be used specifically to identify areas that need improvement and target training or interventions based on these findings.

### Limitations and Solutions

The creators of the scale took into account the previously identified limitations of Likert scale measurements, and ensured that only one subject was asked in one question at a time and that the scale layout was consistent throughout. This scale may be presented electronically by way of survey software and administered through devices such as laptops and smart phones, allowing for greater dissemination and a wider data collection sampling pool.

## Conclusion

The CMCS scale is a successful measure of an individual’s confidence while working in a healthcare setting. It has good internal consistency and validity. It is one of the few confidence scales designed to capture the confidence levels in nursing students when dealing with challenging situations. It can be applied in alternative education settings for the identification of confidence levels in those working or learning in healthcare settings.

## Key points for policy, practice and/or research

- We have created and validated a new scale to measure nursing students’ confidence levels in dealing with challenging situations.
- The Confidence in Managing Challenging Situations Scale (Walsh and Owen, 2018) has been created for use with particular reference to the UK's Nursing and Midwifery Council proficiencies and is based on experiences of students, service users and qualified staff.
- The Confidence in Managing Challenging Situations Scale (Walsh and Owen, 2018) could be adapted to other health student settings.
